# Characterisation of the Binding Properties of *Bacillus Thuringiensis *18 Toxin on Leukaemic Cells

**DOI:** 10.1186/1756-9966-29-86

**Published:** 2010-06-30

**Authors:** Rebecca SY Wong, Shar M Mohamed, Vishna D Nadarajah, Ibrahim Azmi T Tengku

**Affiliations:** 1 Division of Human Biology, School of Medical Sciences, International Medical University. No 126, Jalan 19/155B, Bukit Jalil, 57000 Kuala Lumpur, Malaysia; 2 Department of Preclinical Sciences, Faculty of Veterinary Medicine, University Putra Malaysia, Serdang, 43100 Selangor, Malaysia

## Abstract

**Background:**

Various strains of *Bacillus thuringiensis *(Bt) have been found to produce parasporal proteins that are cytotoxic to human cancer cells. This study aims to establish the binding affinity of purified Bt 18 toxin for CEM-SS (T lymphoblastic leukaemia cell line), to determine if competition exists between the toxin and commercial anticancer drugs for the binding site on CEM-SS and to localise the binding site of the toxin on CEM-SS.

**Methods:**

In homologous competitive binding study, the purified toxin was labelled with biotin and allowed to compete with unlabelled toxin for binding sites on CEM-SS and its dissociation constant (Kd) was determined. Comparisons were made with CCRF-SB, CCRF-HSB-2 and MCF-7. In heterologous competitive binding study, biotinylated toxin competition was determined with two other Bt toxins (crude Btj and crude Bt 22) and anticancer drugs (cisplatin, doxorubicin, etoposide, navelbine and methotrexate). To localise the binding site under the confocal microscope, the biotinylated toxin was tagged with FITC-conjugated streptavidin.

**Results:**

Homologous competitive binding assays revealed decreasing binding affinity of Bt 18 toxin for CEM-SS, CCRF-SB, and CCRF-HSB-2 with Kd of 8.44 nM, 14.98 nM and 17.71 nM respectively. Kd for MCF-7 was not determined as the inhibitory concentration (IC_50_) was not reached. Heterologous competitive study showed little competition (< 30%) between biotinylated Bt 18 toxin and all test compounds used. Confocal microscopy revealed binding of toxin at the periphery of the cell.

**Conclusions:**

It was postulated that purified Bt 18 toxin binds on the cell surface of CEM-SS and the mechanism of cell death may differ from that of Btj toxin, Bt 22 toxin and all five anticancer drugs used in this study, since it did not significantly compete with these compounds for the same binding site.

## Background

*Bacillus thuringiensis *(Bt) is a gram positive, facultative aerobic and spore-forming bacteria. It produces parasporal inclusions containing various insecticidal delta-endotoxins during its sporulative phase and has been used in agricultural fields as an insecticide for decades [1,2]. Recently, it has been found that parasporal proteins of Bt exhibit cytotoxic effect on human cancer cells [3-5]. In 2000, the word parasporin was first introduced by Mizuki *et al*. to describe bacterial parasporal proteins capable of discriminatively killing cancer cells [6]. To date, four classes of parasporins have been identified, namely parasporin 1 (PS1), parasporin 2 (PS2), parasporin 3 (PS3) and parasporin 4 (PS4) [7]. Though many studies have been carried out to characterise these parasporins and to investigate their mechanism of action on human cancer cell lines, little is known about the cancer cell-killing mechanism and the receptors to which these proteins bind on cancer cells. This is especially true for PS3 and PS4 [7]. Previously we demonstrated that purified *Bacillus thuringiensis *(Bt) 18 toxin, from Bt 18, a Malaysian isolate, was selectively cytotoxic against CEM-SS but not human T lymphocytes and was non-haemolytic [8]. We hypothesised that the toxin binds to a specific receptor on CEM-SS and that it competes with commercially available anticancer drugs for the receptor. This study was therefore conducted to further investigate the binding affinity of the toxin for CEM-SS, its interaction with other Bt toxins and commercially available anticancer drugs for binding sites on CEM-SS and to localise where the toxin binds to the cells. Since leukaemia is a common and deadly disease, there is an urgency to develop new and more efficient treatment methods to deal with the problem. Purified Bt 18 toxin used in this study represents a good potential therapeutic agent as it is selectively cytotoxic to CEM-SS, non-cytotoxic to human T lymphocytes and non-haemolytic. These properties of purified Bt 18 toxin may allow it to be used as part of a combination therapy on top of current anticancer drugs, thus lowering the dose required for these drugs. This study shows that purified Bt 18 toxin binds on the cell surface of CEM-SS and its mechanism of cell death may differ from that of Btj toxin, Bt 22 toxin and the selected anticancer drugs since it did not significantly compete with these compounds for the same binding site.

## Methods

### *Bacillus thuringiensis *culture, activation and purification

*Bacillus thuringiensis *was grown to induce sporulation in conditions described by Nadarajah *et al*. [8]. After sporulation Bt was lysed using 1 M NaCl solution and centrifuged at 9000 rpm for 10 mins at 4°C. The pellet was washed once with 1 M NaCl solution, twice with dH_2_O and re-suspended in Tris/KCl buffer (10 mM Tris/HCl, 10 mM KCL, pH7.5). Inclusions were separated from spores by ultracentrifugation at 25,000 rpm, 4°C for 16 hours on a discontinuous sucrose density gradient of 67%, 72% and 79% (w/v) in Tris/KCl buffer as described by Thomas and Ellar [9]. Paraporal inclusions were then solubilised and activated using similar methods as described by Nadarajah *et al*. [8]. The supernatant containing the activated proteins was collected after centrifugation at 13000 rpm for 5 mins at 4°C. The solubilised and activated proteins were desalted using Amicon^® ^Ultra centrifuge tubes (Millipore) with PBS (pH7.4) by centrifugating at 75000 rpm, 4°C for 15 mins. The desalted proteins were purified by means of FPLC using Resource Q™ (Amersham Biosciences) high performance column connected to AKTA™ System. The start buffer used was 20 mM piperazine and the elution buffer, 1 M NaCl. Proteins were separated into 15 ml tubes, concentrated and desalted with PBS (pH7.4).

### Human T lymphocyte extraction

After approval by the ethics committee and informed consent, 20 ml of blood was drawn from a healthy donor. To each ml of whole blood, 50 μl of ResetteSep^® ^Human T Cell Enrichment Cocktail was added and the mixture was incubated at room temperature for 20 mins. The sample was diluted with equal volume of PBS, layered on top of Ficoll-Pague™ Plus in a 15 ml tube and centrifuged for 35 mins at 5000 rpm at room temperature. The enriched T cells found at the Ficoll-Pague™ Plus: plasma interface were aspirated and washed twice with PBS before use.

### Cell culture

Human T lymphocytes, CEM-SS (T-lymphoblastic leukaemic cells), CCRF-SB (B lymphoblasts from acute lymphoblastic leukaemic patient), CCRF-HSB-2 (T lymphoblasts from acute lymphoblastic leukaemic patient) and MCF-7 (breast cancer cells) were cultured using either RPMI 1640 medium (human T lymphocytes, CEM-SS, CCRF-SB and CCRF-HSB-2) or DMEM medium (MCF-7) supplemented with 10% foetal bovine serum, 1% 100 IU/ml penicillin and 100 μg/ml streptomycin, 1% sodium pyruvate and 1% HEPES solution at 37°C in a humidified 5% CO2 atmosphere.

### Determination of protein concentration and sodium dodecyl sulphate polyacrylamide gel electrophoresis (SDS-PAGE) analysis

Protein concentration was determined using the method of Bradford [10]. SDA-PAGE analysis was carried out on the solubilised and activated parasporal proteins as described by Laemmili and Favre [11] and Thomas and Ellar [9] using a 4% (w/v) stacking gel and 10% (w/v) resolving gel.

### Biotinylation of purified Bt 18 toxin and detection of biotinylated toxin

Appropriate volume (calculated using manufacturer's formulae) of 10 mM solution of sulfo-NHS-LC-biotin (Pierce) was added to purified Bt 18 toxin in 1:50 molar ratio and was incubated at 4°C for 2 hours. Excess biotin was removed by size exclusion using Amicon^® ^Ultra centrifugal filter device by centrifugation at 7500 rpm for 10 minutes at room temperature. The biotinylated was detected using the HABA-avidin method. The HABA-avidin solution was prepared by adding 60 μl of 0.01 M HABA (4'-hydroxyazobenzene-2-carboxylix acid) (Pierce) to 1 mg of ImmunoPure^® ^Avidin (Pierce). The solution was then made up to 2 ml using PBS (pH7.4) solution. The HABA-avidin solution was placed in the negative control wells and test wells of a flat-bottom 96-well microplate. Its absorbance was measured at 500 nm. The decrease in absorbance in comparison with the control wells indicated the presence of biotinylated toxin.

### Cell viability assays

Cytotoxic tests were performed as described in previously published literature [8]. Briefly, 50 μl of various concentrations (0 μg/ml to 160 μg/ml) of filtered Bt toxin or anticancer drug was added to 50 μl of exponentially growing cell suspensions (2 × 10^6 ^cells/ml). The treated cells were then incubated at 37°C for 72 hours. The standard MTT ((3-[4,5-dimethylthizol-2-yl]-2,5-diphenyltetrazolium bromide) colorimetric method was applied as described by Shier [12]. Reading of absorbance was carried out at 550 nm with reference at 620 nm. The 50% inhibition concentration (IC_50_) values were deduced from the dose-response curves.

### Homologous competitive binding assays

Fixed concentration (7.41 nM) of biotinylated toxin and increasing concentrations (0 nM to 59.26 nM) of unlabelled purified Bt 18 toxin were added to CEM-SS (2 × 10^6 ^cells/ml) in a 96-well flat bottom microplate. A negative control was also included. The plate was incubated at 37°C for 1 hour. All unbound toxins were removed by centrifugating the microplate at 1200 rpm for 10 minutes at room temperature and the supernatant removed. Detection of the biotinylated purified Bt 18 toxin was by the HABA-avidin method above. Homologous competitive binding assays for other cell lines (CCRF-SB, CCRF-HSB-2 and MCF-7) were carried out in the same manner. The dissociation constant was calculated by determining the IC_50 _(dose at which 50% displacement of the biotinylated purified Bt 18 toxin occurred) and by applying the IC_50 _in the modified Cheng and Prusoff equation [13].

### Heterologous competitive binding assays

Heterologous competitive binding assays were carried out for two different Bt toxins (crude Btj and crude Bt 22 toxins) and five commercially available anticancer drugs (cisplatin, doxorubicin, etoposide, methotrexate, navelbine). Conditions were the same as those used in homologous competitive binding assays.

### Localisation of binding site of purified Bt 18 toxin on CEM-SS

Untreated cells and cells treated with 29.63 nM of biotinylated purified Bt 18 toxin at 1, 2, 12 and 24 hours were fixed using 4% formaldehyde for 15 minutes at room temperature. Upon fixative removal, cell pellets were washed twice using Hank's balanced salt solution (HBSS), re-suspended in HBSS and incubated with 1 μl of ready-to-use fluorescein-conjugated avidin (Pierce), 5 μl of reconstituted Alexa Fluor^® ^594 (Invitrogen) and 2 μl of ready-to-use Hoechst 33342 (Invitrogen) for 15 minutes at room temperature in a dark room. The dyes were removed by centrifugation and the cell pellets were washed twice using HBSS solution and re-suspended in HBSS solution. One drop of the sample (approximately 10 μl) was placed on a microscope slide followed by one drop of ProLong Gold antifade reagent (Invitrogen). The sample was cured for at least 24 hours in the dark before viewing under the confocal microscope (Carl Zeiss).

### Statistical analysis

Statistical analysis was done using SPSS version 16.0. For comparison of two means, the paired t-test was used. P values less than or equal to 0.05 were taken as statistically significant and values less than or equal to 0.001 were taken as highly significant.

## Results

### The effect of biotinylated Bt 18 toxin and the unlabelled toxin on cell viability of CEM-SS

**Figure 1:**
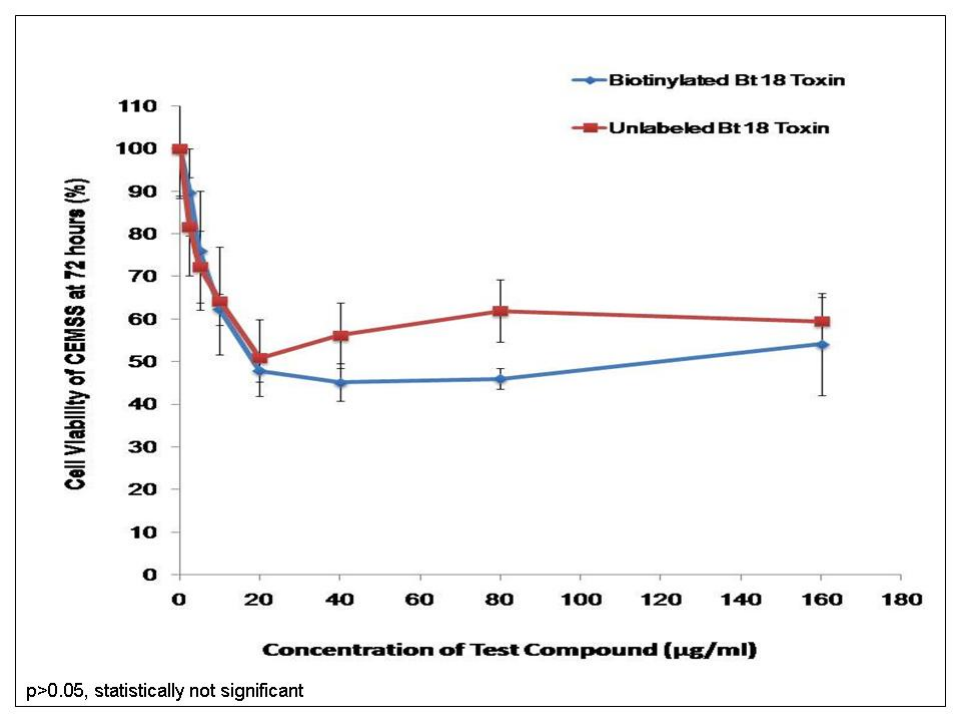
**Cell viability assay-comparing the effect of biotinylated and unlabelled purified Bt 18 toxin on CEM-SS**. Both biotinylated purified Bt 18 toxin and the unlabelled toxin were incubated with CEM-SS cells at 37°C for 72 hours.

Purified Bt 18 toxin had similar effect on CEM-SS at 72 hours whether biotinylated or unlabelled (Figure 1). The highest percentage of cell death achieved by the biotinylated toxin was 45.87% (+/-2.21%) and that of the unlabelled toxin was 40.55% (+/-5.79%). The difference was statistically insignificant (p > 0.05).

### Homologous competitive binding assays

**Figure 2:**
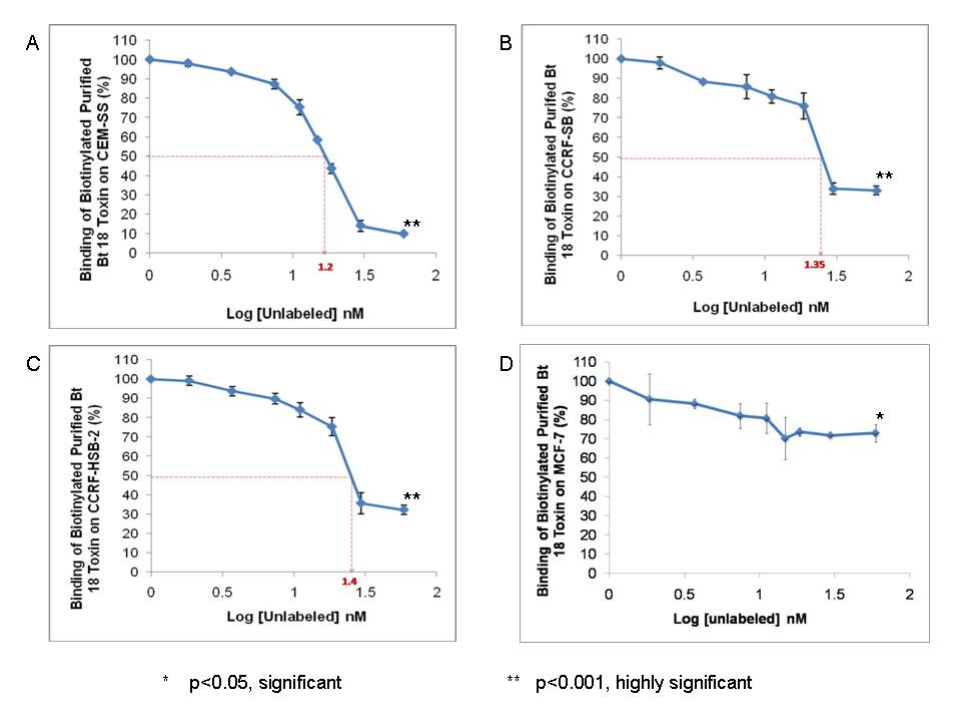
**Homologous competitive binding assays**. The unlabelled toxin and biotinylated purified Bt 18 toxin were allowed to compete for binding site on A) CEM-SS, B) CCRF-SB, C) CCRF-HSB-2 and D) MCF-7 separately using fixed concentration (7.41 nM) of biotinylated purified Bt 18 toxin and various concentrations (0 nM to 59.26 nM) of the unlabelled toxin for 1 hour at 37°C.

Similar trends were observed for CEM-SS, CCRF-SB and CCRF-HSB-2 (Figures 2a, 2b and 2c respectively) i.e., as the concentration of the unlabelled toxin increased, the percentage of the biotinylated purified Bt 18 toxin bound to the cells decreased markedly. However, for MCF-7 (Figure 2d), the decrease in the percentage of the bound biotinylated toxin was not as marked. At 59.29 nM, the unlabelled toxin significantly decreased the percentage of binding of biotinlylated purified Bt 18 toxin on CEM-SS, CCRF-SB, CCRF-HSB-2 and MCF-7 to 9.75%, 33.58%, 33.75% and 72.89% respectively (p < 0.01 for first 3 cell lines, and p < 0.05 for MCF-7). The IC_50 _(concentration at which 50% of the biotinylated purified Bt 18 toxin was displaced) were 15.85 nM, 22.39 nM and 25.12 nM for CEM-SS, CCRF-SB and CCRF-HSB-2 respectively. MCF-7 did not achieve the inhibitory concentration. The Kd was calculated using derivative of the Cheng and Prusoff equation [13]. It was found to be 8.44 nM, 14.98 nM and 17.71 nM for CEM-SS, CCRF-SB and CCRF-HSB-2 respectively. For MCF-7, the dissociation constant could not be determined because the inhibitory concentration was not achieved.

### Heterologous competitive binding assays

**Figure 3:**
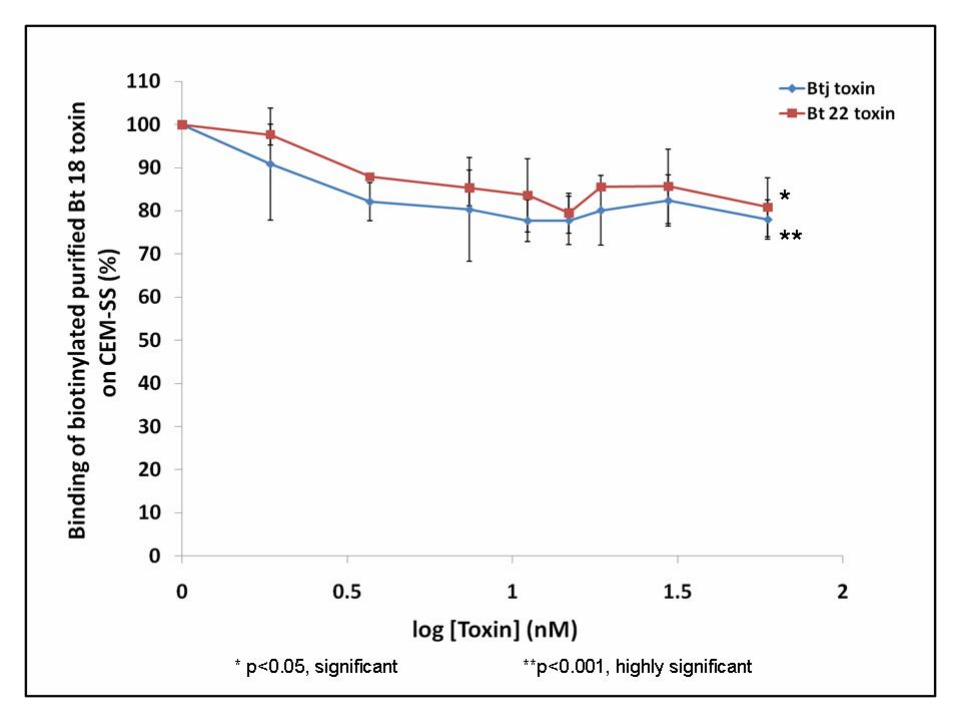
**Heterologous competitive binding assays- biotinylated purified Bt 18 toxin versus crude Btj and crude Bt 22 toxins**. Fixed concentration (7.41 nM) of biotinylated purified Bt 18 toxin was allowed to compete with various concentrations (0 nM to 59.26 nM) of crude Btj toxin and crude Bt 22 toxin separately for 1 hour at 37°C using CEM-SS cell line.

Similar trends were observed for both crude Btj toxin and crude Bt 22 toxin (Figure 3). Both graphs showed a decreasing trend in the percentage of biotinylated purified Bt 18 toxin bound to CEM-SS with increasing toxin concentrations. For crude Btj toxin the percentage of bound biotinylated purified Bt 18 toxin significantly decreased from 100% to 78% at 59.26 nM (p < 0.001). For crude Bt 22 toxin, the percentage of bound biotinylated purified Bt 18 toxin significantly decreased from 100% to 80.81% at 59.26 nM (p < 0.05). However, the difference between crude Btj toxin and crude Bt 22 toxin was statistically insignificant (p > 0.05).

**Figure 4:**
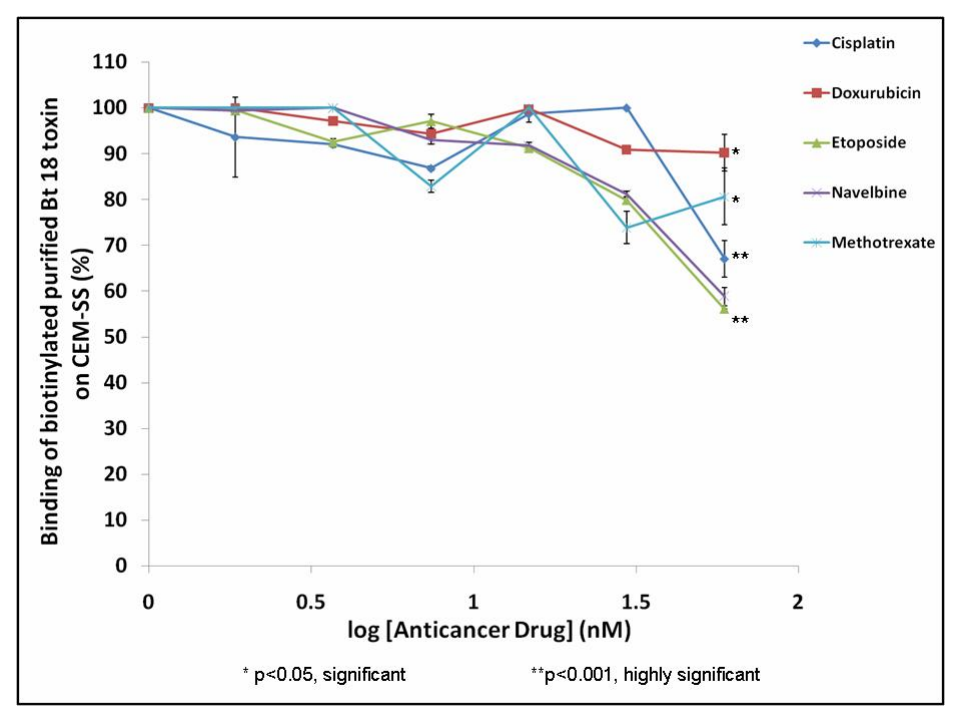
**Heterologous competitive binding assays- biotinylated purified Bt 18 toxin versus various anticancer drugs**. Fixed concentration (7.41 nM) of biotinylated purified Bt 18 toxin was allowed to compete with various concentrations (0 nM to 59.26 nM) of cisplatin, doxorubicin, etoposide, methotrexate and navelbine, separately for 1 hour at 37°C.

It was observed that the graphs show a similar pattern for all the anticancer drugs i.e., the higher the drug concentration, the lower the percentage of biotinylated purified Bt 18 toxin bound to CEM-SS cells (Figure 4). There was a statistically significant but minor decrease in the percentage of binding of the biotinylated toxin on CEM-SS cells when competed with methotrexate (< 30%, p < 0.05) and doxorubicin (< 10%, p < 0.05) with increasing drug concentration. On the other hand, it was found that cisplatin, etoposide and navelbine caused a greater decrease (> 30%) in the percentage of binding of the biotinylated toxin on CEM-SS with increasing drug concentration. This decrease was significant for cisplatin and etoposide (p < 0.001) but insignificant for navelbine (p > 0.05) at the highest drug concentration (59.26 nM). The percentage of displacement of the biotinylated toxin on CEM-SS at the highest drug concentration for the cisplatin, doxorubicin, etoposide, navelbine and methotrexate were 32.76%, 9.82%, 44.67%, 40.27% and 20.40% respectively. However, such high percentage of displacement of the biotinylated toxin at the highest drug concentration was also confounded by a high percentage of cell death (results not shown). Therefore, displacement of the biotinylated toxin at the highest drug concentration may or may not be due to true competition.

### Localisation of the binding site of purified Bt 18 toxin on CEM-SS

**Figure 5:**
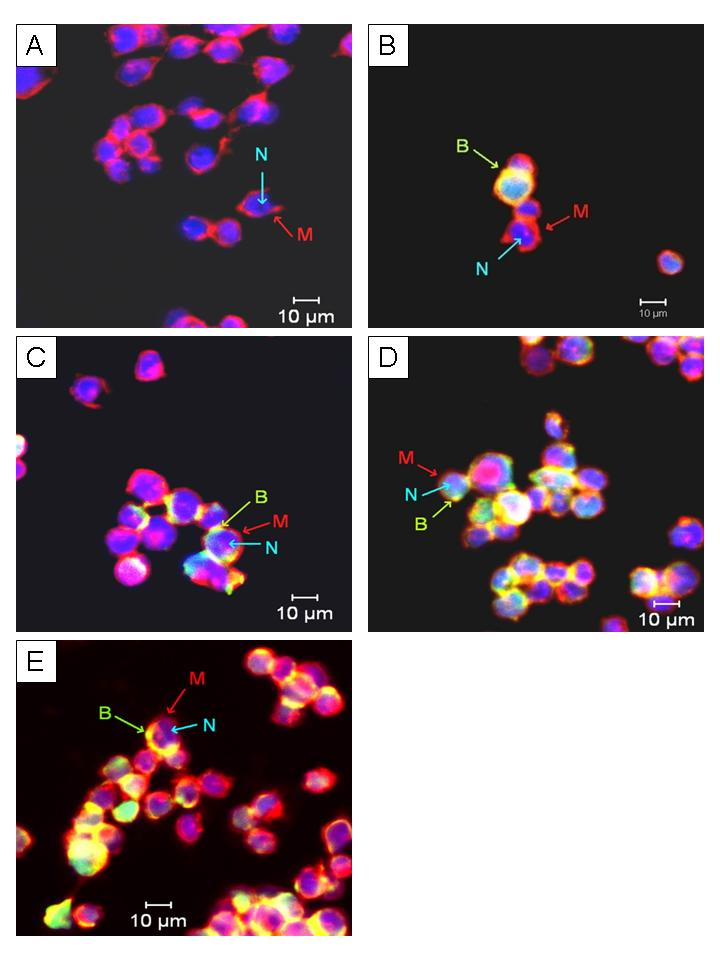
**Confocal microscopic appearance of biotinylated purified Bt 18 toxin-treated CEM-SS**. CEM-SS cells were treated with fixed concentration of biotinylated purified Bt 18 toxin at various time intervals. The biotinylated toxin was then tagged with FITC-conjugated streptavidin (green). The biotinylated toxin was found at the periphery of treated CEM-SS cells. Increased binding of the biotinylated toxin was observed with increased incubation period. (A) Untreated negative control at 24 hours. (B) to (E) Treated cells at 1, 2, 12 and 24 hours respectively. Magnification: 630×. B = Biotinylated purified Bt 18 toxin, M = Cell membrane, N = Nucleus. Bar = 10 μm.

**Figure 6:**
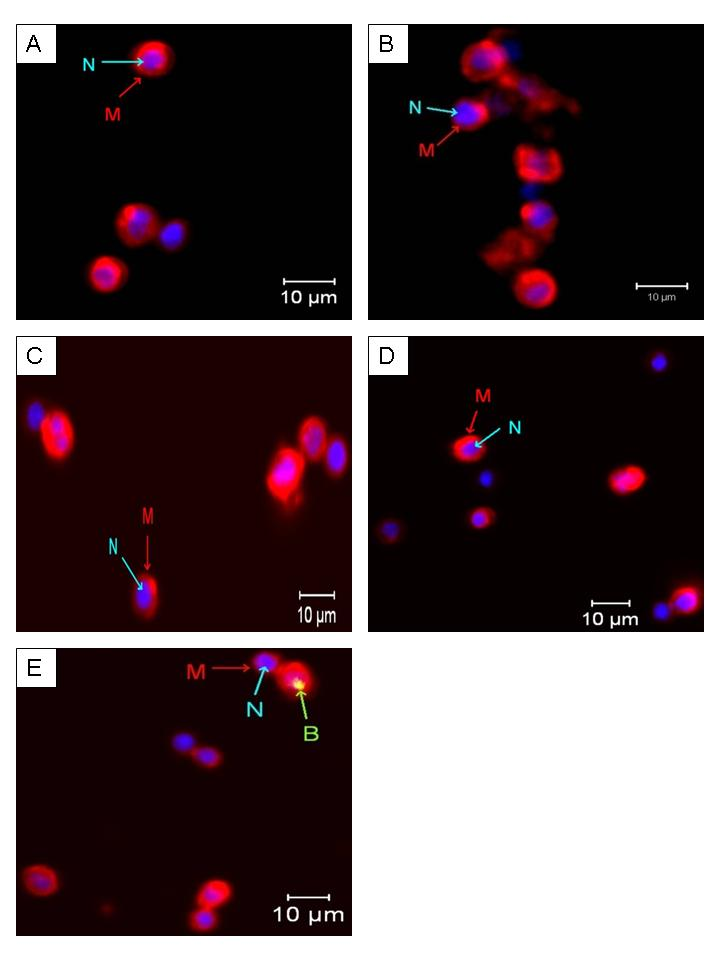
**Confocal microscopic appearance of biotinylated purified Bt 18 toxin-treated human T lymphocytes**. Human T lymphocytes were treated with fixed concentration of biotinylated purified Bt 18 toxin at various time intervals. The biotinylated toxin was then tagged with FITC-conjugated streptavidin (green). Biotinylated toxin was not found at all intervals except 24 hours, where the binding was very minimal. (A) Untreated negative control at 24 hours. (B) to (E) Treated cells at 1, 2, 12 and 24 hours respectively. Magnification: 630 ×. B = Biotinylated purified Bt 18 toxin, M = Cell membrane, N = Nucleus. Bar = 10 μm.

Under the confocal microscope, the cell membrane of CEM-SS and T lymphocytes stained with Alexa Fluor^® ^594 appeared red while the nucleus stained with Hoechst 33342 appeared blue. On the other hand, biotinylated purified Bt 18 toxin tagged with FITC-conjugated streptavidin appeared green or greenish yellow (as a result of overlap with Alexa Fluor^® ^594). For CEM-SS (Figure 5), biotinylated purified Bt 18 toxin appeared at all test intervals (1, 2, 12 and 24 hours). The intensity and extent of staining increased relatively with increased incubation period. The biotinylated toxin was seen at the periphery of the cell where the cell membrane was located. For human T lymphocytes (Figure 6), biotinylated purified Bt 18 toxin did not appear at all test intervals except at 24 hours. Compared to CEM-SS at the same interval, the intensity and extent of staining was very much less remarkable.

## Discussion

Binding studies related to Bt have largely been carried out in insects mainly for characterisation of Bt toxins and the determination of Bt toxin resistance in insects [14]. According to data from cell viability assays in this study, biotinylation did not affect the biological activity of the toxin. This phenomenon was observed in other studies [15,16]. Results from the homologous competitive assays suggested that purified Bt 18 toxin had a higher affinity for CEM-SS, followed by CCRF-SB and CCRF-HSB-2 as the dissociation constant is inversely proportional to the binding affinity. For MCF-7, the dissociation constant could not be obtained because the inhibitory concentration (IC_50_) was not achieved. This could be explained by the fact that CEM-SS, CCRF-SB and CCRF-HSB-2 are all leukaemic cells while MCF-7 represents a very different class of cells, i.e. breast cancer cells. While the first three types may all express specific binding sites for purified Bt 18 toxin, MCF-7, being a totally different class of cells, may not exhibit similar binding sites for the toxin. Since comparisons had already been made between CEM-SS and two other leukaemic cell lines (CCRF-SB and CCRF-HSB-2), MCF-7 was used in this case to demonstrate that a different class of cell line may show lower affinity for the purified toxin. When compared to experiments performed previously, the binding results agreed well with the cell viability assays. Purified Bt18 toxin exhibits cytotoxocity towards CEM-SS cells whereas MCF-7 cells are relatively unharmed [17]. The lower cytotoxicity of the toxin for MCF-7 cells may be explained by the lower affinity the toxin has for these cells.

The scarcity of literature for the binding mechanisms of parasporin makes comparison of binding affinity of purified Bt 18 toxin on CEM-SS with other Bt parasporal proteins and cancer cell types difficult. However, from binding experiments done on insects, it was found that the dissociation constants of various Bt toxins for insect cells were higher than that of purified Bt 18 toxin for CEM-SS cells. As the dissociation constant is inversely proportional to the binding affinity, this implies that binding affinity of purified Bt 18 toxin for CEM-SS cells was relatively higher than that of other Bt toxins for insect cells [18,19]. This finding is interesting as it may mean that the weak cytotoxicity of purified Bt 18 toxin on leukaemic cells could be influenced by factors other than its binding affinity for the cell line since the binding affinity was found to be relatively higher in comparison with insect studies.

Heterologous competitive binding assays suggested that there was a minor degree of competition between biotinylated Bt 18 toxin and crude Btj toxin as well as crude Bt 22 toxin as the percentage of bound biotinylated toxin was significantly decreased to 78% (p < 0.001) and 80.81% (p < 0.05) at 59.26 nM respectively. This low degree of competition might or might not represent true competition among toxins because it was also observed that at such concentration, there was a significant cell death of 10.66% (p < 0.05) and 2.65% (p < 0.05) for crude Btj toxin and crude Bt 22 toxin respectively (results not shown). The decrease in the percentage of the bound biotinylated toxin might be confounded by cell death that occurred at the same time. Besides, it may also be confounded by the possibility of non-specific binding sites. However, even if true competition were to occur, the degree of competition was small as only approximately 20% displacement of the biotinylated toxin occurred for both crude Btj toxin and crude Bt 22 toxin. Little or no competition between biotinylated purified Bt 18 toxin and crude Btj toxin further supported earlier results by Nadarajah *et al*., as purified Bt 18 toxin was non-haemolytic and non-cytotoxic to normal human T lymphocytes while crude Btj toxin was both haemolytic and cytotoxic to normal human T lymphocytes [8].

Heterologous competitive binding assays using anticancer drugs showed that there was a decrease in the percentage of bound biotinylated purified Bt 18 toxin in cell population treated with the anticancer drugs at 59.26 nM (32.76%, 9.82%, 44.67%, 40.27%, 20.40% for cisplatin, doxorubicin, etoposide, navelbine and methotrexate respectively). The selected anticancer drugs in this study bind to and enter cancer cells via various mechanisms and target various sites in these cells. For instance, methotrexate is an antimetabolite and a potent inhibitor of the enzyme dihydrofolate reductase (DHFR) which blocks DNA synthesis and stops cell replication [20]. Navelbine is a vinca alkaloid which binds to tubulin and causes inhibition of the assembly of the mitotic spindles, arresting cells in metaphase and induces apoptosis [21]. Both doxorubicin and etoposide exert their cytotoxic effect by forming a complex with DNA and topoisomerase II, leading to breaks in double-stranded DNA [20,22]. On the other hand, cisplatin works by binding to DNA via intrastrand and interstrand crosslinks. This leads to inhibition of DNA replication and transcription, resulting in breaks and miscoding and eventually apoptosis [20]. By competing purified Bt 18 toxin with each anticancer drug separately, it allows one to study the mechanism of action of purified Bt 18 toxin by comparing with that of the anticancer drug. If the drugs and the toxin showed competition then there is a possibility of these drugs either interfering with toxin binding to CEM-SS cells or the toxin and the drugs sharing a common binding site on the cell membrane which initiates a sequence of events leading to cell death. All results for the competitive binding were statistically significant (p < 0.05) except for navelbine (p > 0.05). However, two confounding factors need to be taken into consideration. Firstly, these findings were confounded by a significantly high percentage of cell death at such high drug concentration (59.26 nM) (21.98%, 11.72%, 22.95%, 22.10%, and 10.92% for cisplatin, doxorubicin, etoposide, navelbine and methotrexate respectively, p < 0.001). Next, there was a possibility of competition for non-specific binding sites on CEM-SS cells. Due to these confounding factors, it was difficult to infer from the results obtained whether the decrease in the percentage of bound biotinylated purified Bt 18 toxin was due to true competition or confounders. However, what could be deduced from the results was that at lower drug concentrations, there was little competition occurring between the toxin and all 5 drugs tested as the percentage of displacement of the biotinylated toxin was small (< 30%), which suggested that the binding sites (hence mechanism of action) might differ for purified Bt 18 toxin and the commercially available anticancer drugs chosen in this study. Although there was little competition between purified Bt 18 toxin and all the five anticancer drugs tested, the heterologous competitive binding assays can be a useful screening tool for drug-toxin interaction and for those drugs that do not compete with purified Bt 18 toxin for binding sites on CEM-SS cells, it may be useful to test for drug synergism in future studies.

Confocal microscopy showed that purified Bt 18 toxin bound to the periphery of CEM-SS cells, suggesting that the binding site could be a cell surface receptor. This finding coincided with immunofluorescent findings of Kitada *et al*. in a study of the cytocidal action of parasporin-2 on cancer cells [23]. It was found that parasporin-2 was distributed at the cell periphery and the immunostaining pattern was the same as the native distribution of cadherin, a cell-cell adhesion protein in the plasma membrane [23]. In addition, increased binding of the biotinylated toxin on CEM-SS cells was observed when the incubation period was increased. The extent of binding was seen to be most remarkable at 24 hours. On the other hand, no biotinylated purified Bt 18 toxin was detected at all test intervals in human T lymphocytes except at 24 hours. Even at 24 hours, the extent of binding on human T lymphocytes was minimal or much less remarkable when compared to CEM-SS cells. Such weak or minimal binding of the purified toxin on human T lymphocytes coincided with the fact that purified Bt 18 toxin did not exert cytotoxic activity on human T lymphocytes.

## Conclusions

In conclusion, purified Bt 18 toxin binds to the periphery of CEM-SS, suggesting that the toxin most likely binds to a cell surface receptor, which is specific to the toxin. It is most likely that purified Bt 18 toxin binds to binding sites that differ from crude Btj toxin or crude Bt 22 toxin. Although confounding factors and limitations were present at high concentrations, at low concentrations of anticancer drugs, there was little competition between purified Bt 18 toxin and the drugs used in this study, suggesting that purified Bt 18 toxin most likely binds to different binding sites on CEM-SS when compared to the anticancer drugs. Hence, the mechanism of action of purified Bt 18 toxin may differ from that of the anticancer drugs used in this study. Such data prompts us to carry out further investigations, such as drug synergism between purified Bt 18 toxin and commercially available anticancer drugs and *in vivo *studies.

## Competing interests

The authors declare that they have no competing interests.

## Authors' contributions

RSYW performed all the experimental tests in this study and participated in data and statistical analysis and writing of this manuscript. SMM participated in experimental design and data analysis. VDN contributed to experimental design, data analysis, editing and submission of this manuscript. TATI participated in data analysis. All authors read and approved the final manuscript.
